# Current Scenario of Clinical Cancer Research in Latin America and the Caribbean

**DOI:** 10.3390/curroncol30010050

**Published:** 2023-01-04

**Authors:** Gustavo Gössling, Taiane F. Rebelatto, Cynthia Villarreal-Garza, Ana S. Ferrigno, Denisse Bretel, Raul Sala, Juliana Giacomazzi, William N. William, Gustavo Werutsky

**Affiliations:** 1 Latin American Cooperative Oncology Group (LACOG), Porto Alegre 90619-900, RS, Brazil; 2 Breast Cancer Center Hospital Zambrano Hellion TecSalud—Tecnologico de Monterrey, Monterrey 66278, NL, Mexico; 3 Grupo de Estudios Clínicos Oncológicos del Perú (GECO PERU), Lima 15038, Peru; 4 Grupo Argentino de Investigación Clínica en Oncología (GAICO), Rosario S2124KBO, Argentina; 5 Hospital BP—Beneficência Portuguesa de São Paulo, São Paulo 01323-001, SP, Brazil

**Keywords:** cancer, clinical trials, Latin America, Caribbean

## Abstract

In Latin America and the Caribbean (LAC), progress has been made in some national and regional cancer control initiatives, which have proved useful in reducing diagnostic and treatment initiation delays. However, there are still significant gaps, including a lack of oncology clinical trials. In this article, we will introduce the current status of the region’s clinical research in cancer, with a special focus on academic cancer research groups and investigator-initiated research (IIR) initiatives. Investigators in LAC have strived to improve cancer research despite drawbacks and difficulties in funding, regulatory timelines, and a skilled workforce. Progress has been observed in the representation of this region in clinical trial development and conduct, as well as in scientific productivity. However, most oncology trials in the region have been sponsored by pharmaceutical companies, highlighting the need for increased funding from governments and private foundations. Improvements in obtaining and/or strengthening the LAC cancer research group’s financing will provide opportunities to address cancer therapies and management shortcomings specific to the region. Furthermore, by including this large, ethnic, and genetically diverse population in the world’s research agenda, one may bridge the gap in knowledge regarding the applicability of results of clinical trials now mainly conducted in populations from the Northern Hemisphere.

## 1. Introduction

By 2030, 75% of all cancer deaths will occur in low- and middle-income countries (LMICs), with one in eight people experiencing a cancer diagnosis in their lifetime [1]. Most Latin American and Caribbean countries, which comprise 8.4% of the world’s population, are considered middle-income countries [2,3,4]. In 2020, according to GLOBOCAN, of the total of 19.3 million estimated new cancer cases, 1.4 million were registered in Latin America and the Caribbean. This growing cancer incidence in already overburdened and underresourced settings is concerning, given existing disparities in healthcare access and coverage [5,6,7].

In Latin America and the Caribbean, progress in cancer control has been made, as national and regional initiatives on cancer screening, patient navigation programs, and telemedicine have proved helpful in reducing diagnostic and treatment initiation delays. However, there are still critical gaps, including more investment in large programs for cancer control, non-universal health coverage, fragmented health systems, the inequitable concentration of cancer services, delays in diagnosis or treatment initiation, limited access to molecular and genomic tests, inadequate access to new targeted drugs, insufficient palliative services, registry inefficiency, scarcity of reliable data, and lack of clinical trials in oncology [7,8,9].

Cancer research is heavily skewed toward high-income countries, with disproportionately fewer studies conducted in and relevant to the problems of LMICs, including Latin America and the Caribbean [10,11]. For example, of all randomized clinical trials (any phase) evaluating cancer therapies conducted worldwide from 2014 to 2017, only 8% (58 of 694) were available in low- and middle-income countries. These data demonstrate that trials are concentrated in high-income countries and do not appropriately represent the global burden of cancer. Importantly, the trial results are not necessarily generalizable across populations and country contexts. Studies also suggest funding and publication bias against randomized controlled trials led by LMICs. In addition, they highlight that trials from these countries are more likely to identify effective therapies and have a more significant effect size than trials from high-income countries [11,12,13].

Strengthening research capacity at the individual, organizational, network, and policy levels is essential for long-term benefits and sustainability. Randomized clinical trials remain the most powerful tool for changing clinical practice and improving outcomes. Thus, efforts must focus on translating research into care and policies that support and drive widespread quality care delivery as a key initiative in oncology [14,15,16]. Thus, we discuss the current status of cancer clinical trials developed in Latin America and the Caribbean, the academic cancer research groups working in these regions, and investigator-initiated research (IIR) initiatives conducted by these teams.

## 2. Current Status of Cancer Clinical Trials in Latin America and the Caribbean

The challenges to the participation of Latin America and the Caribbean in clinical trials investigating cancer therapies are determined by several factors, including the lack of public and private investment, scarcity of local funding, and delays in research regulatory processes. In this article, we will now outline all these challenges [7,12].

First, the low level of investment in cancer research is a significant barrier to local initiatives investigating tailored strategies in Latin American countries. The percentage of gross domestic product (GDP) spent on research and development during 2008–2011 in Latin American countries was 0.65%, varying from 1.16 in Brazil, 0.6 in Argentina, 0.4 in Mexico, 0.37 in Chile, 0.16 in Colombia, and 0.15 in Peru. This is 3.4 times less than in developed countries, such as South Korea, Japan, the USA, and Germany, with GDP rates of 3.74, 3.36, 2.9, and 2.8, respectively. The lack of regional investment hampers drug development in these nations and poses a challenge in developing tailored treatment strategies based on local epidemiologic data [17,18].

The limited number of cancer specialists and the lack of formal training in research procedures can cripple participation in clinical trials. For example, Mathew et al. [19] reported that the ratio of clinical oncologists per new cancer diagnosis in high-middle income South American countries ranges from 1:170 in Brazil, 1:287 in Argentina, 1:331 in Peru, to 1:667 in Chile, which compares unfavorably to the ratio of 1:137 found in the United States. For Brazil, the largest country in Latin America, Portich et al. [20] reported rates of 1:541 in 2014 and 1:342 in 2020 of Brazilian medical oncologists with registration in the Council of Clinical Cancerology, not including hematologists and radiotherapists, per new cancer diagnosis in these years. In addition, despite the increasing number of medical oncologists in Brazil related to the growth of residencies and fellowship positions compared to 2014, the distribution of medical oncologists in different Brazilian regions remains heterogeneous [19].

Foreign donors and sponsors largely dictate the research agenda because of a scarcity of local funding [21]. Most governmental grants available in Latin America and the Caribbean are insufficient to maintain competitive research endeavors [22]. In Mexico, for example, researchers enrolled in the National Research System received monthly stipends of ~USD 380–1790 in 2018 [23]. This stipend would be insufficient to cover standard article processing charges (~USD 2600) [24] and research activities if not supplemented by additional funding. Noteworthy, 6.1% of medical oncologists from Latin America and the Caribbean declared that they were the main sponsor of their research proposals in 2009–2010, with up to 41.2% Mexican and 25% Peruvian physicians stating that they supported their projects with their resources [25].

Additionally, scientists’ salaries in the region are suboptimal, and medical doctors often prefer to pursue careers more focused on clinical practice than research [22]. Those who choose to become physician-scientists are often overwhelmed with clinical duties and are not provided with protected time for conducting research [16]. Another point to address is the frequent delays in the research regulatory process. Most Latin American and Caribbean oncologists declared that it might take 3 to 6 months to obtain study approval, with 9.8% stating that it requires more than a year [25].

Despite these barriers to performing clinical trials, Latin America and the Caribbean are still very attractive regions for research. Latin America and the Caribbean unite unique cancer epidemiology, a high population density, a large ethnic and genetic diversity of the population, relatively low costs for most procedures, and a high potential for patient recruitment [7,12]. Globalization of clinical trials has increased the participation of Latin America and the Caribbean countries but did not result in significant increases relative to global numbers.

In 2022, 4167 (5.5%) clinical trials, of a total of 75,084 trials on cancer treatments registered in clinicaltrials.gov involved Latin America and Caribbean countries. Among these, 1673 (40.1%) are recruiting, not yet recruiting, or active. Out of the total of the trials in Latin America and the Caribbean (n: 4168): n: 183 (4.4%) were phase I trials, 976 (23.4%) were phase 2 trials, 2722 (65.3%) were phase 3 trials, and 140 (3.3%) were phase 4 trials. The countries most cited as participating sites were Brazil (1349 trials; 32.3%), Argentina (766 trials; 18.3%), Mexico (751; 18.0%), Chile (424 trials; 10.2%), and Peru (328 trials; 7.9%) [26]. An average of 83% of the studies in Latin America and the Caribbean are sponsored by industry, 15% are sponsored by individuals, universities, and organizations, and 2% by the National Institutes of Health (NIH) and other US Federal Agencies [26] (Table 1).

## 3. Search Strategy and Selection Criteria

No formal systematic review was conducted. The list of all the countries that comprise Latin America and the Caribbean was obtained from the World Bank [27]. We conducted an updated literature search focused on the current scenario of cancer clinical trials in Latin America and the Caribbean, including public and private investments, local funding, and research regulatory processes.

Information regarding the number of clinical trials in these regions was accessed on the webpage of the National Institutes of Health (NIH) U.S. National Library of Medicine (Clinical Trials) on 28 October 2022 [28]. In the “advanced search” it was included the “name of each Latin America/Caribbean country” in the field “location > country”. In “condition or disease,” “cancer” was included, and in “study type,” we selected “interventional studies (clinical trials).” The number of scientific publications from these regions was searched on PubMed on 28 October 2022, for the last five years, using this search strategy: “neoplasms” [MeSH Terms], “clinical trial” [All Fields], “name of each LATAM/Caribbean country” [All Fields], and using the filter of “publication data” >5 years [29]. The incidence and mortality rates were accessed on GLOBOCAN using the tool “Cancer Today” [1]. The main Academic Cancer Research Groups were those who had more scientific publications in Latin America and the Caribbean. The investigator-initiated research of these academic groups, its infrastructure, and considerations about challenges, advantages, and opportunities for clinical research in this region were obtained by means of a questionnaire delivered to each group.

The fact that there are few phases I and II studies and also few academic studies in Latin America and the Caribbean can be associated with the lack of public investments and an adequate infrastructure for supporting clinical sites to conduct studies following Good Clinical Practice, timely and regulatory requirements [30]. Pharmaceutical companies sponsor most clinical trials. According to a search of the International Clinical Trials Registry and the Clinical Trials Platform, the number of sponsored clinical trials has increased in these regions over the last decade. Fortunately, it provides greater access to new cancer therapies for populations for whom up-to-date treatments are unavailable [30,31,32,33].

A steady rise in scientific publications in Latin America and the Caribbean in oncology and cancer research has been observed in recent years (from 1978 citable documents published in 2015 to 3410 in 2020) [30]. Brazil was the most prolific country in 2020, with 1561 citable documents (45.8%), followed by Mexico (676 documents; 19.8%) and Argentina (270 documents; 7.9%). However, despite recent advances, the scientific productivity of the region is still lagging far behind Northern America (33,710 citable documents published in 2020) and Western Europe (44,416 documents) [34].

## 4. Academic Cancer Research Groups and Investigator-Initiated Research in Latin America

**Figure 1:**
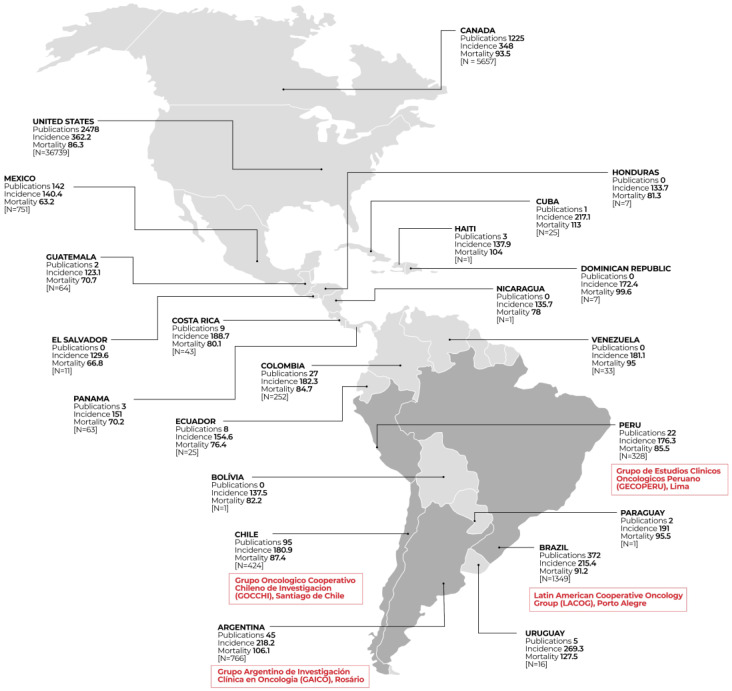
Headquarters of collaborative groups working in Latin America and the Caribbean, cancer incidence and mortality, and the number of cancer clinical trials (N) per country.

Worldwide, several collaborative and academic cancer research groups work to prevent, detect, and treat cancer. Most of these groups are in the Northern Hemisphere and Australia [12,35]. However, some collaborative groups work in Latin America and the Caribbean, focusing on solid tumors, which are described below and in Figure 1:

**The Latin American Cooperative Oncology Group (LACOG)** is a non-profit organization founded in 2009 in Porto Alegre, Brazil. This is Latin America’s first multinational cooperative group dedicated to clinical and translational cancer research. LACOG currently counts more than 400 investigator members in 194 institutions from 16 Latin American countries. (https://lacogcancerresearch.org (accessed on 28 October 2022)).

**Grupo Argentino de Investigación Clínica en Oncología (GAICO)** is a cooperative group of specialists dedicated to oncology and developing and designing clinical trials in Argentina. (https://www.gaico.org.ar (accessed on 28 October 2022)).

**Grupo de Estudios Clinicos Oncológicos Peruano (GECOPERU)** is a group founded in 2005 in Peru that works to develop cancer research in the fields of basic sciences, epidemiology, translational research, and clinical trials.

**Grupo Oncologico Cooperativo Chileno de Investigacion (GOCCHI)** is a cooperative research group that promotes collaboration between Chilean cancer centers. It was founded in Chile in 1998 by a group of specialists. (https://gocchi.org (accessed on 28 October 2022)).

**The Latin American Consortium for the Investigation of Lung Cancer (CLICAP)** is a consortium funded in 2011 that works to improve lung cancer research in Latin America.

Table 2 describes the studies carried out by the four main Academic Cancer Research Groups in Latin America and the Caribbean, their infrastructure, and considerations about challenges, advantages, and opportunities for clinical research in this region. All four groups develop epidemiological and clinical studies and have a research structure set up to conduct studies. The groups with the highest volume of studies are GOCCHI and LACOG, and most of the studies are sponsored by the pharmaceutical industry on breast and lung cancers. The challenges, advantages, and opportunities for clinical research cited by these groups comprehensively demonstrate the weaknesses and strengths of conducting clinical trials in the region.

These academic cancer research groups develop investigator-initiated research (IIR), which involves studies conceived, developed, and sponsored by independent investigators, institutions, or collaborative/cooperative groups. Resources, including funding and drug product supplies to develop IRR trials, are provided by pharmaceutical companies, government programs, fundraising campaigns, and self-funding by investigators or institutions. IIR are extremely important to generate data on the effectiveness and safety of a drug in the real-world setting and attempt to answer questions that clinicians face in their day-to-day practice and to be in line with the aspiration to improve global access to medicines, a key opportunity in developing countries from Latin America and the Caribbean [30,36].

All teams from these groups reported that a workforce would need to be focused on optimizing regulatory processes. It will make the region more attractive to sponsors and, consequently, will improve recruitment to clinical trials in these regions and retention of highly qualified cancer professionals and researchers. Additionally, the competitive advantages of Latin America were reinforced, such as the high population density, the high willingness of patients to accept participating in clinical trials, and the lack of language barriers.

Despite the slightly increasing participation of countries in clinical trials in recent years, ranging from 6.04% in 2015 to 8.54% in 2021, the number of IIR projects remains low in these regions. The main reasons for the low representativeness of these countries in IIR include limited financial resources, along with challenges in regulatory submissions and well-trained study personnel, such as data managers, statisticians, medical monitors, and technical writers [36]. Additionally, investigators must go through a competitive process to reach grants through the IIR programs offered by the pharmaceutical industry. Thus, investigators from Latin America and the Caribbean compete with investigators across the world, which limits their chances of being chosen.

There are limited or no government research grants in Latin America and the Caribbean. This reflects the little attention these countries give to research and the low proportion of GDP destined for research. Nevertheless, some initiatives from countries such as Brazil have provided alternatives. For example, PRONON (Programa Nacional de Apoio à Atenção Oncológica) was a program that provided funds to non-profit private institutions, associations, and foundations to expand oncology assistance, support training, and develop clinical and epidemiological studies. However, a few projects were approved because of the limited spending ceiling that could be used in this program, and the project expired in 2021 [35].

Figure 1 demonstrates the location of the main four Academic Cancer Research Groups in Latin America and the Caribbean countries and highlights the cancer incidence and mortality of each country, the number of clinical trials, and publications related to cancer in the National Library of Medicine (National Institutes of Health 2022).

Brazil has the highest number of cancer clinical trials (*n* = 1349) and scientific publications about cancer clinical trials (372 in the last five years) and high rates of cancer incidence and mortality (215.4 and 91.2 per 100,000 individuals, respectively). In second place is Argentina, with 766 cancer clinical trials and higher incidence and mortality rates (218.2 and 106.1 per 100,000 individuals, respectively). However, with a small number of publications when compared to countries such as Chile, which has a higher number of publications (*n* = 95), a lower number of clinical trials (*n* = 424), and lower incidence and mortality rates (180.9 and 87.4 per 100,000 individuals). In third place in terms of the number of cancer clinical trials is Mexico, with 751 cancer clinical trials and 142 publications; however, the incidence rates are not among the highest in the region (140.4 per 100,000 individuals), and its mortality rates are low (63.2 per 100,000 individuals).

Countries such as Uruguay, Cuba, and Paraguay have expressive incidence and mortality rates; however, they conduct a low number of clinical trials (16, 25, and 1, respectively).

## 5. Future Directions

Although significant progress has been made in the representation of Latin America and the Caribbean in the development and conduct of clinical trials, as well as in scientific productivity, considerable challenges remain. Countries seeking to facilitate cancer research would benefit from improving available funding and ensuring that researchers have protected time for their endeavors.

With an increasing burden over the next decades in Latin America and the Caribbean, there is a need to plan investments in cancer treatment [37]. It is essential to consider that structural reforms in healthcare systems, new programs for disenfranchised populations, expansion of cancer registries, and implementation of policies are needed to improve this region’s primary and secondary cancer care. However, these regions are working hard in cancer research despite drawbacks and difficulties in funding, regulatory timelines, a skilled workforce, and well-trained study personnel.

The academic Cancer Research Groups working in this region have an essential role in training medical oncologists and research specialists in proposing investigator-initiated studies evaluating topics with epidemiological importance for this region. In addition, the increasing participation in multicentric studies presented by pharmaceutical industries demonstrates the possibility of streamlined logistics and rapid, high-volume recruitment in this region. Pharmaceutical companies have funded most oncology trials in Latin America and the Caribbean, and increased funding will also be needed from government or private foundations.

By including the large, ethnic, and genetically diverse populations of Latin America and the Caribbean in the world’s research agenda, one may bridge the gap in knowledge regarding the applicability of the results of clinical trials evaluating new cancer treatments now mainly conducted in populations from the Northern Hemisphere.

## Figures and Tables

**Table 1:** Cancer clinical trials conducted in Latin America and the Caribbean.

Country	Clinical Trials	Study Phase								Status		Funder Type					
		Early Phase 1/Phase I		II		III		IV		Completed	Not Yet Recruiting/Recruiting /by Invitation/Active Not Recruiting	Industry		All Others (Individuals, Universities, Organizations)		Others (Nih, Other US Federal Agencies)	
Brazil	1349	63	5.2%	350	28.7%	759	62.3%	47	3.9%	605	529	964	71.2%	371	27.4%	18	1.3%
Argentina	766	42	5.3%	190	24.1%	536	68.1%	19	2.4%	368	319	719	93.7%	42	5.5%	6	0.8%
Mexico	751	31	4.2%	176	23.8%	505	68.4%	26	3.5%	329	307	627	83.3%	117	15.5%	9	1.2%
Chile	424	27	6.3%	89	20.6%	302	70.1%	13	3.0%	180	211	386	90.6%	35	8.2%	5	1.2%
Peru	328	9	2.8%	79	24.5%	227	70.3%	8	2.5%	188	104	277	84.2%	18	5.5%	34	10.3%
Colombia	252	7	2.9%	36	15.0%	189	78.8%	8	3.3%	124	111	224	88.9%	22	8.7%	6	2.4%
Guatemala	64	0	0.0%	10	16.1%	49	79.0%	3	4.8%	28	29	61	95.3%	2	3.1%	1	1.6%
Panama	63	2	3.1%	11	17.2%	49	76.6%	2	3.1%	40	13	60	95.2%	3	4.8%	0	0.0%
Costa rica	43	0	0.0%	7	15.9%	34	77.3%	3	6.8%	13	29	40	93.0%	0	0.0%	3	7.0%
Venezuela	33	0	0.0%	3	9.7%	22	71.0%	6	19.4%	24	2	30	90.9%	2	6.1%	1	3.0%
Cuba	25	0	0.0%	17	63.0%	10	37.0%	0	0.0%	12	7	19	76.0%	6	24.0%	0	0.0%
Ecuador	25	1	5.6%	1	5.6%	13	72.2%	3	16.7%	18	5	18	72.0%	7	28.0%	0	0.0%
Uruguay	16	0	0.0%	2	12.5%	12	75.0%	2	12.5%	14	0	14	87.5%	2	12.5%	0	0.0%
El salvador	11	0	0.0%	2	25.0%	6	75.0%	0	0.0%	7	4	8	72.7%	0	0.0%	3	27.3%
Honduras	7	0	0.0%	2	33.3%	4	66.7%	0	0.0%	5	2	5	62.5%	1	12.5%	2	25.0%
Dominican Republic	7	0	0.0%	0	0.0%	4	100.0%	0	0.0%	6	1	6	85.7%	0	0.0%	1	14.3%
Bolívia	1	1	50.0%	1	50.0%	0	0.0%	0	0.0%	1	0	1	100.0%	0	0.0%	0	0.0%
Nicaragua	1	0	0.0%	0	0.0%	1	100.0%	0	0.0%	0	0	0	0.0%	1	100.0%	0	0.0%
Paraguay	1	0	0.0%	0	0.0%	1	100.0%	0	0.0%	1	0	1	100.0%	0	0.0%	0	0.0%
Haiti	1	0	0.0%	0	0.0%	0	0.0%	0	0.0%	0	0	0	0.0%	1	100.0%	0	0.0%
**TOTAL**	**4168**																

Due to overlap or missing data, sums of “Study Phase” and “Funder Type” categories do not necessarily equal the total number of trials or 100%. Source on 21 October 2022 [25].

**Table 2:** Studies conducted by the main academic cancer research groups in Latin America and their infrastructure and considerations about challenges, advantages, and opportunities for clinical research in this region.

Variable	Group (n)
Epidemiological Studies	
Ongoing	GAICO (4); GOCCHI (1); Gecoperu (6); LACOG (14)
Completed	GAICO (0); GOCCHI (0); Gecoperu (4); LACOG (9)
Investigator-initiated	GAICO (4); GOCCHI (0); Gecoperu (4); LACOG (21)
Sponsored by industries	GAICO (4); GOCCHI (1); Gecoperu (6); LACOG (21)
Intergroup contributions	GAICO (4); GOCCHI (1); Gecoperu (2); LACOG (3)
**Clinical Trials**	**Group (n)**
Ongoing/Completed	GAICO (11); GOCCHI (20); Gecoperu (4); LACOG (20)
Investigator-initiated	GAICO (0); GOCCHI (5); Gecoperu (0); LACOG (13)
Sponsored by industries	GAICO (11); GOCCHI (3); Gecoperu (3); LACOG (4)
Intergroup contributions	GAICO (7); GOCCHI (12); Gecoperu (1); LACOG (1)
**Infrastructure of Clinical Research**	**Description (%)**
Areas in oncology	Breast (100%), Radiation (50%), Kidney (50%), Lung (75%), Colon (25%), Pancreatobiliary (25%), Upper GI (Esophageal or Gastric) (25%), Cervical (50%), Prostate (25%), Ovarian (25%), Lymphoma (25%), Head and Neck (25%)
Process executed (without subcontracting third parties)	Ethical Process (100%), Regulatory Process (100%, Pharmacovigilance (75%), Monitoring activities and Data Management (100%), Site contractualization (100%), Site Payments (100%), CRF development (75%), Statistics (25%)
Positions applied to employees	Project Manager (100%), DM and Monitor (100%), CRA (100%), Medical Team (100%), Legal Assistant (50%), IT Support (75%), Statistician (25%)
**Challenges, Advantages, and Opportunities for Clinical Research**	**Description**
Main challenges	Regulatory timelines, political instability, population trust in the pharmaceutical industry, disparity in access to standard therapies, costs, lack of human resources, cultural diversity, lack of protected time for clinical investigators, competing for industry-driven trials
Competitive advantages/opportunities	Availability of trained health professionals, high level of trust from patients (high willingness to participate in clinical trials), relative lack of language barriers (continent with only two dominant languages), genetically diverse population, lack of access to innovative or high-cost therapies, high population density (and increased ease of access to investigator and patients), availability of health professionals, large market, presence of rare diseases
Actions that could further reinforce	Aim to develop academic trials including prevalent tumors of underdeveloped countries such as cervix cancer, support for independent academic research to address questions that are meaningful for the local population (i.e., de-escalating strategies, cost-effective treatments)

## Data Availability

Not applicable.
